# Delayed Type Hypersensitivity Reaction Induced By Liraglutide With Tolerance to Semaglutide

**DOI:** 10.1210/jcemcr/luae105

**Published:** 2024-06-21

**Authors:** Ricardo Moreno-Borque, Guillermo Guhl-Millán, Sara Mera-Carreiro, Mario Pazos-Guerra, Jose Antonio Cortés-Toro, Eduardo López-Bran

**Affiliations:** Dermatology Department, Hospital Clínico San Carlos, 28040 Madrid, Spain; Dermatology Department, Hospital Clínico San Carlos, 28040 Madrid, Spain; Department of Endocrinology and Nutrition, Hospital Clínico San Carlos, 28040 Madrid, Spain; Department of Endocrinology and Nutrition, Hospital Clínico San Carlos, 28040 Madrid, Spain; Department of Anatomic Pathology, Hospital Clínico San Carlos, 28040 Madrid, Spain; Dermatology Department, Hospital Clínico San Carlos, 28040 Madrid, Spain

**Keywords:** semaglutide, GLP-1, delayed hypersensitivity, allergy, obesity

## Abstract

Liraglutide is a glucagon-like peptide-1 (GLP-1) receptor agonist used for the management of type 2 diabetes and obesity. It was the first GLP-1 receptor agonist to be approved by the US Food and Drug Administration and the European Medicines Agency for the treatment of obesity. To date, numerous skin adverse reactions to liraglutide have been reported, but data regarding hypersensitivity reactions are scarce, raising concerns about its safety and clinical management. We present the case of a 56-year-old female patient with class 3 obesity who was started on subcutaneous liraglutide (Saxenda) by her endocrinologist. One month after starting the aforementioned treatment, the patient presented well-defined, round, erythematous pruriginous plaques surrounding the injection site, around 24 hours after the drug administration. A liraglutide-induced, delayed-type hypersensitivity reaction was suspected, which could be subsequently confirmed by allergy testing and histopathological study. This paper explores the clinical use of liraglutide, the occurrence of hypersensitivity reactions, diagnosis, management, and implications for future research. Understanding and managing liraglutide hypersensitivity is crucial to ensuring the safety and efficacy of this medication.

## Introduction

Liraglutide is a glucagon-like peptide-1 (GLP-1) receptor agonist that has gained popularity for its effectiveness in managing type 2 diabetes and obesity, becoming one of the top treatments according to different diabetes guidelines [1]. Its mechanism of action involves stimulating insulin secretion, inhibiting glucagon secretion, slowing gastric emptying, and reducing food intake, making it a valuable tool in diabetes and obesity management. Liraglutide, marketed under the brand name Saxenda (Novo Nordisk), has demonstrated significant benefits in glycemic control, weight loss, and cardiovascular outcomes in diabetic patients [2]. It is typically administered as a once-daily subcutaneous injection in the lower abdomen, thighs, or back of the arms, and is a part of the treatment regimen for many individuals with diabetes.

A variety of hypersensitivity reactions to liraglutide have been reported in the current medical literature [3‐7]. These reactions can manifest in various ways, including skin rashes, itching, swelling, gastrointestinal symptoms, respiratory distress, and even anaphylaxis. Hypersensitivity reactions can range from mild to severe, potentially posing a substantial risk to patients. We have the impression that these previously reported cases may underscore the clinical significance of liraglutide hypersensitivity.

Our case report provides a thorough overview of “liraglutide hypersensitivity,” covering clinical use, previous published experiences, diagnosis, management, and future considerations. It serves as a resource for health-care professionals, researchers, and anyone interested in the intersection of obesity management and hypersensitivity reactions to liraglutide.

## Case Presentation

We present the case of a 56-year-old female patient whose comorbidities included prediabetes, hypertension, hypertriglyceridemia, class 3 obesity, allergic rhinitis, and gastroesophageal reflux disease. The patient reported an allergy to penicillin that had never been confirmed. Her regular medication comprised inhaled ipratropium bromide, bilastine, intranasal budesonide, paracetamol, candesartan/hydrochlorothiazide, and omeprazole. All of her medications were part of chronic treatments, and no recent changes in them were reported. On physical examination, her height was 152 cm, body weight 93 kg, body mass index was 40 (class 3 obesity), and blood pressure 150/91 mm Hg. Glucose level was measured as 111 mg/dL (6.2 mmol/L) (normal reference range, 70-99 mg/dL; 3.9-5.5 mmol/L), glycated hemoglobin 6.2% (normal reference range, 4%-5.7%), thyrotropin 1.85 µIU/mL (normal reference range, 0.4-4 µIU/mL), low-density lipoprotein 138 mg/dL (3.6 mmol/L) (normal reference range, 100-129 mg/dL; 2.6-3.3 mmol/L), high-density lipoprotein 55 mg/dL (1.42 mmol/L) (normal reference range, 40-60 mg/dL; 1.0-1.6 mmol/L), total cholesterol 267 mg/dL (6.92 mmol/L) (normal reference range, < 200 mg/dL; < 5.2 mmol/L), and triglycerides 495 mg/dL (5.58 mmol/L) (normal reference range, < 150 mg/dL; < 1.69 mmol/L). The rest of the analytical parameters were within normal limits.

**Figure 1.**
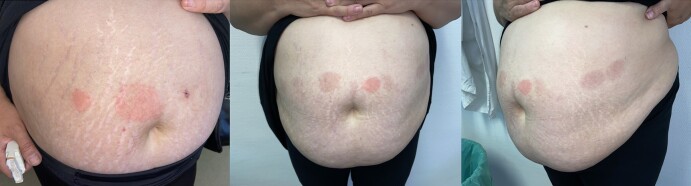
Well-defined, round erythematous plaques surrounding the injection site of liraglutide.

Her endocrinologist started her on subcutaneous liraglutide (Saxenda) as a first-line treatment for her obesity. She initially started at a once-daily subcutaneous dose of 0.6 mg, with weekly increments of 0.6 mg until a dose of 1.8 mg/day was achieved. One month after starting the daily 1.8 mg subcutaneous dose, the patient gradually developed well-defined, round erythematous pruriginous plaques (55 × 45 mm) surrounding the injection site, around 24 hours after the drug administration (Fig. 1). At no point was there mucosal involvement or any systemic symptoms associated.

With this clinical picture, a delayed-type hypersensitivity reaction induced by liraglutide was suspected and the patient was referred by her endocrinologist to our dermatology department for assessment.

## Diagnostic Assessment

**Figure 2.**
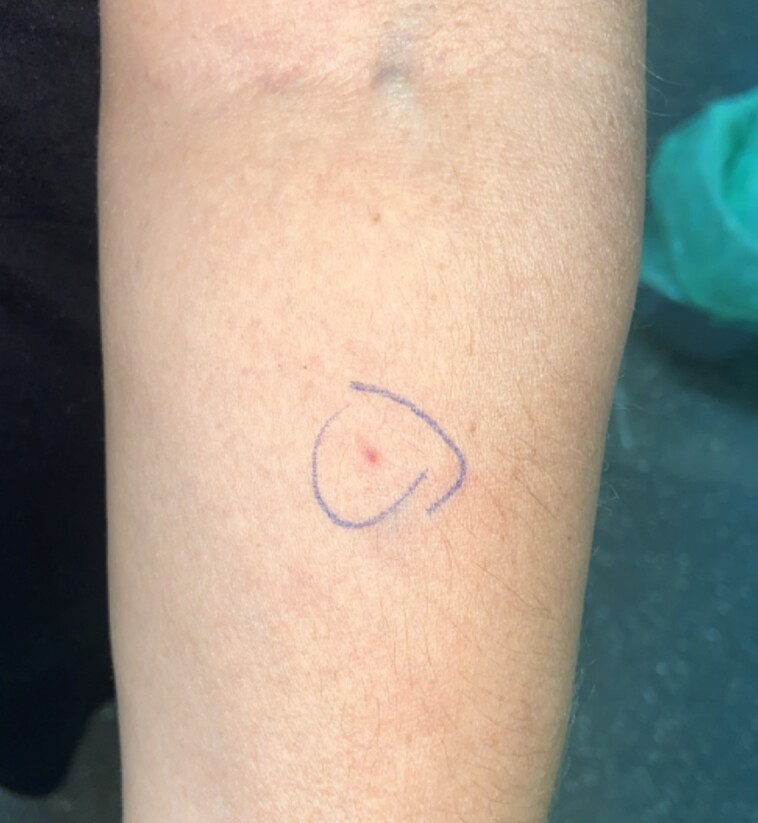
A positive delayed reaction of intradermal testing to liraglutide with 1/1 dilution.

A skin prick test with liraglutide (6 mg/mL; aqueous solution) was performed 2 months after the reaction, and immediate and delayed readings (96 hours) yielded negative results. Intradermal testing (IDT) was performed, and was negative at the 1/1000 (0.006 mg/mL), 1/100 (0.06 mg/mL), and 1/10 (0.6 mg/mL) dilutions [8]. The 1/1 (6 mg/mL) dilution was without question positive after the 24-hour delayed reading, causing a 24 × 29-mm wheal (Fig. 2). An IDT with the 1/1 (6 mg/mL) dilution was performed in 5 healthy controls to rule out a possible irritant reaction due to the concentration used. All control tests yielded negative results both in the immediate and delayed readings.

**Figure 3.**
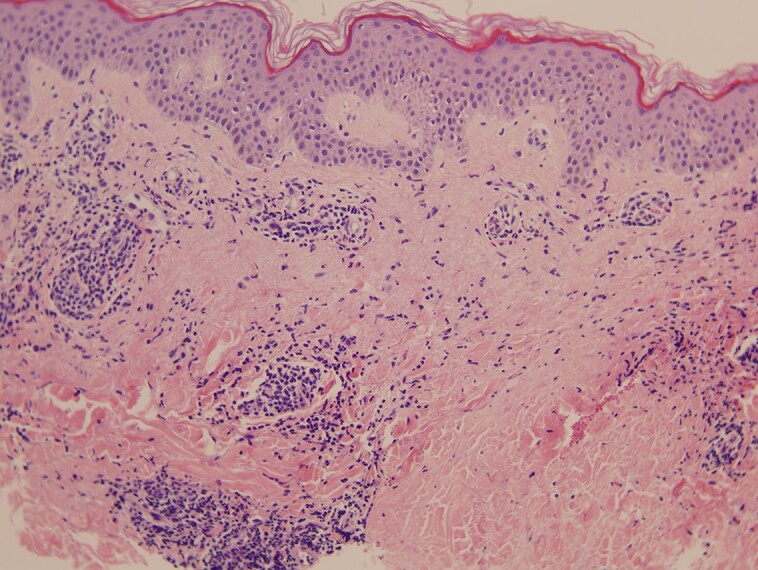
Histology showing no notable alterations in the epidermis and a moderate perivascular inflammatory infiltrate composed mainly of lymphocytes and isolated eosinophils in the dermis.

A skin biopsy was obtained, and histology revealed no notable alterations in the epidermis, whereas in the dermis a moderate perivascular inflammatory infiltrate composed mainly of lymphocytes and isolated eosinophils was found. No dysplasia or evidence of malignancy was observed in the specimen. The skin biopsy findings were compatible with a hypersensitivity cutaneous drug reaction (Fig. 3).

A blood analysis was performed for which blood cell counts, as well as the liver and renal function tests, were normal.

Based on the clinical presentation, and given the results of the complementary tests we performed, the patient was diagnosed with a delayed hypersensitivity to liraglutide, and the drug was discontinued.

## Treatment

The patient was prescribed symptomatic treatment with twice-daily topical betamethasone dipropionate ointment (0.5 mg/g) with partial remission of the lesions, and was called for close follow-up.

## Outcome and Follow-up

**Figure 4.**
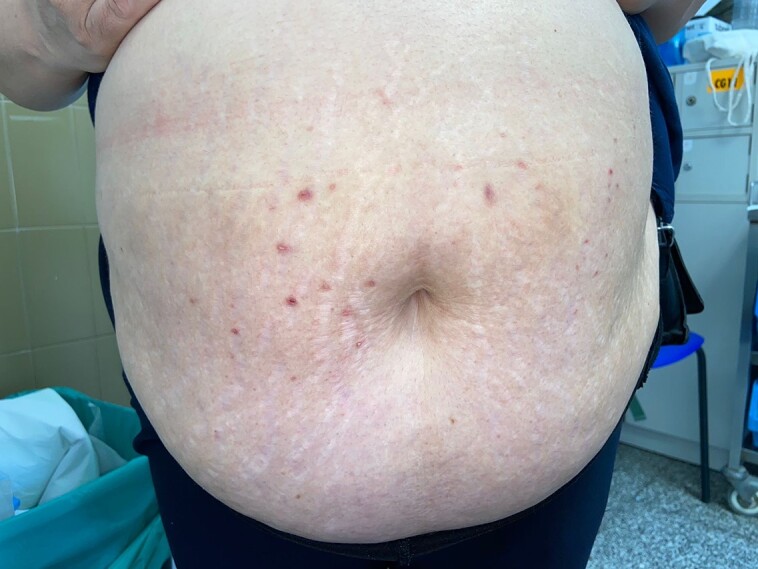
Small, postinflammatory hyperpigmented papules at the sites where the initial lesions were, 6 weeks after treatment cessation.

Five weeks after the cessation of liraglutide, the patient experienced a noticeable improvement of the lesions. The eruption resolved completely 6 weeks after discontinuation of the treatment, leaving small, postinflammatory hyperpigmented papules at the sites where the initial lesions were (Fig. 4).

We proposed semaglutide as an alternative treatment to liraglutide. Both skin prick test (1.34 mg/mL) and IDT (1/100, 1/10, and 1/1 dilutions) to semaglutide yielded negative results. After assessment of risk, a drug provocation test was performed with semaglutide, for which no immediate or delayed reactions were reported. The patient was started on a once-weekly subcutaneous 0.25 mg dose of semaglutide, which was incremented to 0.5 mg/week after 4 weeks of treatment. To date the patient has had no side effects, ruling out cross-reactivity between liraglutide and semaglutide.

## Discussion

The introduction of new GLP-1 receptor agonist drugs results in better treatment of diabetes and obesity, but also in a potential risk of inducing new adverse reactions that have not yet been properly described [9]. Diagnosing liraglutide hypersensitivity can be challenging, as its symptoms can overlap with other allergic reactions or adverse effects of the medication. It is crucial to take a detailed patient history and perform appropriate diagnostic tests [10] to confirm hypersensitivity and differentiate it from other reactions.

Preventing liraglutide hypersensitivity is essential, and health-care providers should be vigilant when prescribing liraglutide, particularly in patients with a history of drug allergies. If hypersensitivity reactions occur, prompt intervention is necessary, which may include a close follow-up, discontinuing liraglutide, and administering treatments such as antihistamines, corticosteroids, or epinephrine in case of severe reactions.

Liraglutide is administered as a once-daily subcutaneous injection in the lower abdomen, thighs, or back of the arms. Although they are generally well tolerated, the most common side effects related with the use of GLP-1 receptor agonists are gastrointestinal (especially nausea, vomiting, and diarrhea). These effects are usually transient, and tend to diminish over time [2]. To reduce the risk of the aforementioned side effects, treatment is usually started with small doses that are gradually increased. Headaches, nasopharyngitis, and bronchitis have been reported quite often, as well. Other more serious side effects have been described with the use of liraglutide such as pancreatitis, thyroid malignancy, anaphylaxis, or severe hypoglycemia [11]. As liraglutide is a peptide, it has the potential to be immunogenic. Approximately 8.6% of patients developed antibodies to liraglutide during the Liraglutide Effect and Action in Diabetes (LEAD) trials [12]. These antibody titers were not associated with an increased risk of developing adverse reactions [13], and this could be explained by the degree of sequence identity between liraglutide and human GLP-1, which is around 97% [14]. Liraglutide molecules self-associate into heptameters in the subcutis, thereby delaying its absorption from the injection site [15]. This fact could trigger T-cell stimulation by liraglutide antigens, thus favoring the development of delayed hypersensitivity reactions [16].

In the Satiety and Clinical Adiposity—Liraglutide Evidence in nondiabetic and diabetic individuals (SCALE) obesity and prediabetes trials [17], the most common cutaneous reaction that patients presented with was injection-site hematoma (5.7%). Other site reactions included deep nodular infiltrate and pruritic erythematous macules. Cutaneous allergic reactions to the drug were all very infrequent (<1%) and mild.

Skin adverse reactions to liraglutide have been rarely reported in the literature. A case of generalized erythematous nodules and plaques with peripheral blood eosinophilia [4], an acute exanthematous pustulosis in photo-exposed areas [7], and a vesiculopustular rash [9] associated with the use of liraglutide have been described.

To our knowledge, only 2 cases of delayed hypersensitivity to liraglutide have been published to date with diagnostic confirmation by means of positive skin tests [3, 5]. Our case is the first one that adds a compatible pathological result in a skin biopsy.

Future research should focus on better understanding the mechanisms of liraglutide hypersensitivity, including the roles of immunological and nonimmunological factors. Improved diagnostic tools, predictive markers, and preventive strategies need to be developed. Additionally, health-care professionals must be educated about the identification and management of liraglutide hypersensitivity.

In conclusion, we report a rare case of a delayed hypersensitivity reaction to liraglutide with positive diagnostic allergy testing and a compatible histopathological result in the skin biopsy. The negative allergy testing and tolerance to semaglutide suggest that this drug could represent a safe therapeutic alternative in patients with allergy to liraglutide.

## Learning Points

- Liraglutide is a valuable tool in diabetes and obesity management, but hypersensitivity reactions pose a considerable clinical challenge.
- Understanding the mechanisms, diagnosis, and management of liraglutide hypersensitivity is critical for ensuring patient safety and effective treatment.
- It is believed that both immunological and nonimmunological factors may contribute to these reactions.
- Semaglutide may be a safe therapeutic alternative in patients with hypersensitivity reactions to liraglutide, despite the structural similarity they share.
- Future research is needed to establish a clear pattern of cross-reactivity between GLP-1 receptor agonists.

## Contributors

All authors made individual contributions to the authorship of this manuscript. R.M.B., G.G., S.M., M.P., and E.L. were involved in the diagnosis and management of the patient and manuscript submission. J.A.C. performed histopathology sectioning and preparation of histology images. All authors reviewed and approved the final draft.

## Data Availability

Data sharing is not applicable to this article as no data sets were generated or analyzed during the current study.

## Abbreviations

GLP-1: glucagon-like peptide-1
IDT: intradermal testing
